# A novel single-tube LAMP-CRISPR/Cas12b method for rapid and visual detection of zoonotic *Toxoplasma gondii* in the environment

**DOI:** 10.1186/s40249-024-01266-5

**Published:** 2024-12-10

**Authors:** Yao Liang, Shi-Chen Xie, Yi-Han Lv, Yuan-Hui He, Xiao-Nan Zheng, Wei Cong, Hany M. Elsheikha, Xing-Quan Zhu

**Affiliations:** 1 Laboratory of Parasitic Diseases, College of Veterinary Medicine, Shanxi Agricultural University, Taigu, 030801 Shanxi People’s Republic of China; 2 Marine College, Shandong University, Weihai, 264209 Shandong People’s Republic of China; 3 Faculty of Medicine and Health Sciences, School of Veterinary Medicine and Science, University of Nottingham, Sutton Bonington Campus, Loughborough, LE12 5RD UK

**Keywords:** *Toxoplasma gondii*, Loop-mediated isothermal amplification, CRISPR/Cas12b, Lateral flow immunoassay, Environmental detection, Zoonotic parasites, Molecular diagnostics

## Abstract

**Background:**

*Toxoplasma gondii* oocysts, excreted in cat feces, pose a significant health risk to humans through contaminated soil and water. Rapid and accurate detection of *T. gondii* in environmental samples is essential for public health protection.

**Methods:**

We developed a novel, single-tube detection method that integrates loop-mediated isothermal amplification (LAMP), the clustered regularly interspaced short palindromic repeats (CRISPR)/Cas12b system, and lateral flow immunoassay strips for rapid, visual identification of *T. gondii*. This method targets the *T. gondii B1* gene*,* initially amplifies it with LAMP, directed by a single-guide RNA (sgRNA). It then recognizes the amplified target gene and activates trans-cleavage, cutting nearby single-stranded DNA (ssDNA) reporters. Fluorescence detection was performed using a 6-Carboxyfluorescein (FAM)-12N-Black Hole Quencher-1 (BHQ1) reporter, while Fluorescein Isothiocyanate (FITC)-12N-Biotin enabled visual detection on lateral flow strips. The method was tested for its ability to detect various *T. gondii* genotypes and related parasites, assessing its specificity and broad-spectrum applicability. It was further applied to real-world environmental samples to evaluate its practicality.

**Results:**

The LAMP-CRISPR/Cas12b method exhibited high specificity and broad-spectrum detection capability, successfully identifying nine *T. gondii* genotypes and distinguishing them from 11 other parasitic species. Sensitivity testing at both molecular (plasmid) and practical (oocyst) levels showed detection limits of 10  copies/μL and 0.1 oocyst, respectively. When applied to 112 environmental samples (soil, water, and cat feces), the method demonstrated 100% sensitivity, accurately reflecting known infection rates.

**Conclusions:**

This LAMP-CRISPR/Cas12b single-tube method offers a robust, innovative approach for monitoring zoonotic *T. gondii* in environmental samples, with significant implications for public health surveillance.

**Graphical Abstract:**

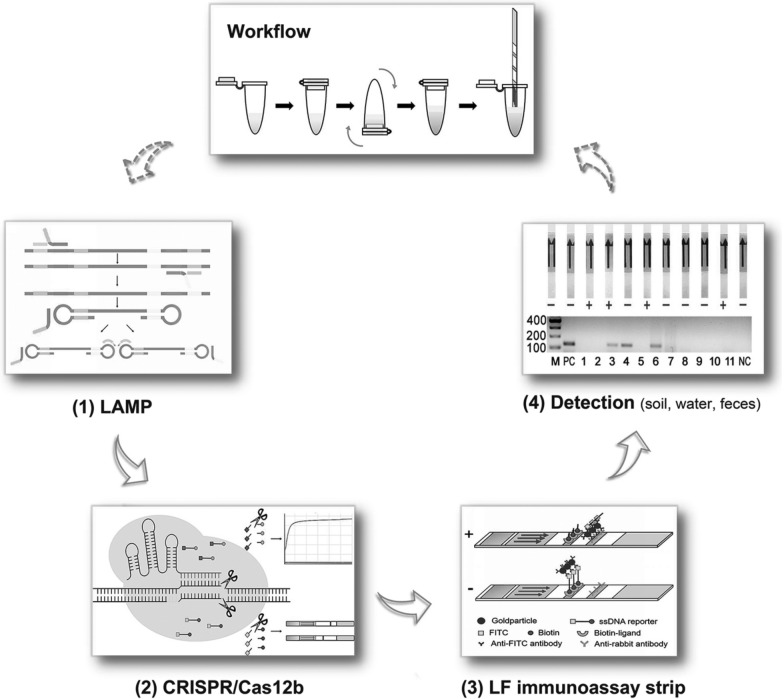

**Supplementary Information:**

The online version contains supplementary material available at 10.1186/s40249-024-01266-5.

## Background

*Toxoplasma gondii* is an intracellular parasitic protozoan capable of infecting a wide range of warm-blooded animals and humans [1]. In immunocompromised individuals, such as those with HIV/AIDS or undergoing immunosuppressive therapy, toxoplasmosis can lead to severe complications, including encephalitis and other life-threatening conditions [2, 3]. It is estimated that approximately one-third of the global population has been infected with *T. gondii*, with prevalence rates varying significantly by region [4, 5]. Global data on acute toxoplasmosis outbreaks show that 44.1% of cases are linked to oocysts, with 20.6% from contaminated water, 17.6% from contact with sand and soil, and 5.9% from consuming contaminated fruits and vegetables [6].

*Toxoplasma gondii* oocysts are shed into the environment through the feces of felines, which are the only definitive hosts for this parasite. Serological studies show a global prevalence of 35% in domestic cats, with wild felines exhibiting an even higher prevalence of 59% [7]. Infected felines can release millions to tens of millions of oocysts [8, 9]. Under favorable environmental conditions, these oocysts develop into highly infectious sporulated forms that can persist for months or even years in moist soil [10]. These characteristics highlight the need for effective environmental monitoring to control and prevent the spread of toxoplasmosis, underscoring the demand for rapid and efficient detection methods for *T. gondii* in environmental samples.

Several methods for detecting *T. gondii* in the environment have been reviewed in recent studies [11, 12], including polymerase chain reaction (PCR) [13, 14], quantitative real-time PCR (qPCR) [15, 16], nested PCR (nPCR) [17, 18], and mouse bioassays [19, 20]. However, each method has its limitations. PCR is widely used but vulnerable to inhibitors in complex environmental samples, such as soil and feces, leading to false negatives and reduced sensitivity [21]. Although qPCR offers enhanced sensitivity and specificity, its high cost and the need for specialized equipment limit its use in resource-limited settings [22]. nPCR, while highly sensitive, is complex, prone to contamination, and has long processing times [23]. The mouse bioassay, traditionally used for *T. gondii* detection, provides direct infection data but is limited by long experimental durations, high costs, and ethical concerns [24]. As a result, there is a clear need for a novel detection method that can rapidly and accurately identify *T. gondii* in various environmental samples to improve monitoring efficiency.

Loop-mediated isothermal amplification (LAMP) is a rapid nucleic acid amplification technique that requires only a DNA polymerase with high strand displacement activity and a set of specific primers, enabling amplification of target genes under isothermal conditions [25, 26]. Previous research has shown that LAMP effectively detects *T. gondii B1*, *SAG1*, and *SAG2* genes, demonstrating excellent sensitivity and specificity with a detection limit as low as 0.1 tachyzoite and no cross-reactivity with other parasites [27].

The CRISPR/Cas system, consisting of clustered regularly interspaced short palindromic repeats (CRISPR) and associated proteins (Cas), is a powerful genomic engineering tool [28, 29]. It has been widely applied in molecular diagnostics [30–32], gene editing [33, 34], metabolic engineering [35], and gene function exploration [36]. The CRISPR/Cas system also holds significant promise for pathogen detection in environmental monitoring, including *T. gondii* detection [37]. Cas12b, a recent addition to the Cas protein family, amplifies detection signals by cleaving non-specific single-stranded DNA (ssDNA) reporters. It also offers high temperature stability and a compact size, making it an ideal tool for molecular diagnostics [38].

The SHERLOCK detection method, which combines recombinase polymerase amplification with CRISPR/Cas13a, has demonstrated enhanced sensitivity compared to using Cas13a alone [39]. Similarly, researchers have combined LAMP with CRISPR/Cas12b to develop the LAMP-CRISPR/Cas12b method, which improves detection sensitivity. This method has been applied to detect various pathogens, including the monkeypox virus [40], *Mycobacterium tuberculosis* [41], and SARS-CoV-2 [42], across fields such as disease monitoring, environmental health, and food safety.

In this study, we focused on the *T. gondii B1* gene, designing LAMP primers and a single-guide RNA (sgRNA), and developed corresponding LAMP and CRISPR/Cas12b detection systems. By combining these two technologies into a single tube format, we developed a LAMP-CRISPR/Cas12b detection method. Detection is possible via a real-time fluorescence quantitative analyzer or visually using lateral flow immunoassay strips, each utilizing specific ssDNA reporters. We evaluated the method’s broad-spectrum applicability and specificity by detecting various *T. gondii* genotypes and distinguishing them from other parasitic species. Comprehensive sensitivity assessments were conducted at both the molecular (plasmid) and practical (oocyst) levels. Additionally, this LAMP-CRISPR/Cas12b single-tube method was successfully applied to environmental samples, providing an efficient tool for monitoring *T. gondii* in the environment and identifying potential transmission pathways.

## Methods

### Preparation of DNA samples

The *B1* gene of the *T. gondii* RH strain (GenBank Accession No. AF179871) was amplified using PCR and the specific primers (forward primer 5′-TGTTCTGTCCTATCGCAACG-3′, reverse primer 5′-ACGGATGCAGTTCCTTTCTG-3′), yielded a 580 bp DNA fragment [43]. The amplified DNA bands were separated through electrophoresis and purified using the StarPrep DNA Gel Extraction Kit (GenStar, Beijing, China). The purified DNA was ligated into the pMD18-T vector (Takara, Dalian, China) to construct recombinant plasmids. These plasmids were then transformed into Trans5α chemically competent cells (TransGen, Beijing, China) and cultured. Plasmid DNA was subsequently extracted using the StarPrep Fast Plasmid Mini Kit (GenStar, Beijing, China) and used as a standard positive control in subsequent experiments.

### LAMP system

LAMP is a rapid, isothermal method for nucleic acid amplification that utilizes a DNA polymerase with strong strand-displacement activity and a specific set of primers targeting multiple regions of the target gene [25, 26]. To optimize the amplification process, both primer design and reaction temperatures were meticulously evaluated. Five sets of LAMP primers were designed to amplify the recombinant plasmid of the *T. gondii B1* gene using Primer Explorer V5 software (http://primerexplorer.jp/e/) and synthesized by Sangon Biotech Co. Ltd (Shanghai, China). Primer sequences are detailed in Additional file 1: Table S1. Before use, the primers were diluted to specific concentrations: FIP and BIP to 16 µmol/L, F3 and B3 to 2 µmol/L, and Loop F and Loop B to 4 µmol/L. The LAMP system was prepared with the WarmStart® Fluorescent LAMP Kit (New England Biolabs, Ipswich, USA), consisting of the following components: 12.5 µL of WarmStart LAMP 2× Master Mix, 0.5 µL of fluorescent dye (50×), 2.5 µL of LAMP primer mix, 2 µL of target DNA, and 7.5 µL of ddH_2_O. The reaction was initially performed at 65 °C. To determine the optimal reaction temperature, additional experiments were conducted at 45 °C, 50 °C, 55 °C, 60 °C, 65 °C, and 70 °C. Fluorescence values were monitored using the QuantStudio 5 Real-Time PCR System (Thermo Fisher Scientific, Waltham, USA) to identify the most effective primers and optimize the reaction conditions.

### CRISPR/Cas12b-based detection system

The CRISPR/Cas12b system utilizes sgRNA to recognize and bind specific DNA sequences, initiating targeted cleavage activity [38]. To construct the pUC18-sgRNA-spacer plasmid, the spacer sequence of sgRNA was incorporated into the pUC18 plasmid (Sangon Biotech, Shanghai, China), with detailed sequences provided in Additional file 1: Table S1. PCR amplification was performed using this plasmid as a template, with the upstream primer T7-sgRNA-F and a downstream primer designed to match the target DNA sequence (Additional file 1: Table S1). The PCR products were visualized through 1% agarose gel electrophoresis, and the desired DNA bands were recovered using the QIAquick Gel Extraction Kit (QIAGEN, Hilden, Germany). These purified DNA fragments served as templates for in vitro sgRNA synthesis using the Transcriptaid T7 Transcription Kit (Thermo Fisher Scientific, Waltham, USA). Three sgRNAs were designed and tested to identify the most effective candidate for guiding the CRISPR/Cas12b detection system.

The CRISPR/Cas12b detection system was assembled in a 20 μL reaction volume with the following components: 1 × HOLMES Buffer 1, 250 nmol/L AacCas12b Nuclease, 250 nmol/L sgRNA, 250 nmol/L target DNA, and 250 nmol/L ssDNA reporter. The ssDNA reporter was labeled with a fluorophore (6-Carboxyfluorescein, FAM) at the 5′ end and a quencher (Black Hole Quencher-1, BHQ1) at the 3′ end (5′–3′: FAM-NNNNNNNNNNNN-BHQ1). Upon cleavage of the reporter, a fluorescence signal was generated and monitored using the QuantStudio 5 Real-Time PCR System. To determine the optimal reaction conditions, the initial reaction temperature was set at 48 °C. Further optimization experiments were conducted across six temperature gradients ranging from 45 to 70 °C, with each reaction lasting 30 min.

### LAMP-CRISPR/Cas12b single-tube method

To minimize aerosol contamination and streamline the detection process, a single-tube method integrating LAMP with Cas12b mediated trans-cleavage was developed. This approach requires precise adjustments to prevent interference between components.

The procedure begins with preparing the LAMP reaction mixture in a PCR tube. The mixture includes 5 µL of WarmStart LAMP 2× Master Mix, 1 µL of LAMP primer mix, 1 µL of target DNA, and 3 µL of nuclease-free water. The total volume of 10 µL was overlaid with 20 µL of mineral oil to insulate heat and prevent contamination. The CRISPR/Cas12b detection system was prepared separately and introduced into the tube cap to prevent premature activation. This system comprised 1 µL of AacCas12b Nuclease (10 µmol/L), 1.5 µL of sgRNA (10 µmol/L), 2 µL of 10 × HOLMES Buffer 1, and 0.5 µL of Recombinant RNase Inhibitor (4 U/µL). For fluorescence detection, 1 µL of FAM-12N-BHQ1 reporter (10 µmol/L) was included. For lateral flow immunoassay, 0.2 µL of Fluorescein Isothiocyanate (FITC)-12N-Biotin reporter (10 µmol/L) was added. The total volume of this detection system was adjusted to 20 µL with nuclease-free water.

The tube was heated to 55 °C for 20 min to allow LAMP reaction. Extended heating times were used for samples with low DNA concentrations. After mixing and a brief centrifugation step, the reaction was heated for an additional 10 min to activate the CRISPR/Cas12b system. Fluorescence signals were measured using the QuantStudio 5 Real-Time PCR System. For lateral flow immunoassay, 70 µL of HybriDetect Assay Buffer was added to the tube, mixed thoroughly and the lateral flow test strip was inserted vertically. Results were visible within 3 min. This method efficiently combines LAMP and CRISPR/Cas12b detection in a single-tube setup, ensuring a contamination-free and streamlined workflow. Its effectiveness was validated using the *T. gondii* RH strain as a positive control and nuclease-free water as a negative control.

### Assessment of broad-spectrum capability, specificity and sensitivity of the LAMP-CRISPR/Cas12b single-tube method

The LAMP-CRISPR/Cas12b single-tube method was evaluated for its broad-spectrum detection, specificity, and sensitivity using a rigorous set of tests. For broad-spectrum detection capability, the method was tested with ten standard *T. gondii* strains, representing nine distinct genotypes: RH, GT1, TgCatBr5, CTG, TgCgCa1, PTG, TgCatBr64, TgRsCr1, MAS, and PYS.

To assess specificity, the method was tested against DNA from 11 non-target parasites, including *Neospora caninum*, *Cryptosporidium andersoni*, *Trypanosoma evansi*, *Trypanosoma brucei*, *Trypanosoma equiperdum*, *Leishmania infantum*, *Leishmania donovani*, *Eimeria tenella*, *Blastocystis* sp., *Enterocytozoon bieneusi*, and *Toxocara canis*.

Sensitivity was evaluated at both molecular and practical levels. Molecular sensitivity was determined using tenfold serial dilutions of plasmid DNA, with concentrations ranging from 1 × 10^5^ to 1 × 10⁻^1^ copies/μL. Extended LAMP times were employed for samples with low DNA concentrations to enhance detection.

To evaluate the assay’s sensitivity in real-world conditions, DNA was extracted from purified *T. gondii* oocysts and serially diluted to concentrations of 100, 10, 1, 0.1, and 0.01 oocysts. To simulate environmental scenarios, the oocysts were spiked into environmental samples confirmed to be free of *T. gondii*. Extended LAMP times were again applied to improve detection at lower oocyst concentrations. To validate the sensitivity of the LAMP-CRISPR/Cas12b single-tube method, gradient-diluted oocyst samples were analyzed concurrently using semi-nPCR [44].

### Detection of environmental samples

The single-tube method was employed to detect *T. gondii* in a variety of environmental samples, including 30 soil samples, 50 water samples, and 32 cat fecal samples. The 30 soil samples were collected from parks, farms, and beaches across six provinces in China [13]. These samples were previously tested by conventional PCR, semi-nPCR, and nPCR, which targeted the 529 bp-repeat element (RE), the *B1* gene, and the multicopy internal transcribed spacer-1 (ITS-1) region of rDNA, respectively. The results revealed that 14 of the soil samples were tested positive for *T. gondii* [13].

The 50 water samples were collected from the Sow River Basin in Weihai, Shandong Province, during a previous study [45]. Of these, 30 samples tested positive for *T. gondii* by nPCR targeting the *B1* gene [45]. Thirty-two cat fecal samples were collected as part of our study on cat immunization with live-attenuated *T. gondii* strains (unpublished data). Eleven of these samples were collected from cats prior to infection and tested negative for *T. gondii* by microscopy and semi-nPCR targeting the *B1* gene. The remaining 10 samples, collected from infected cats, tested positive for *T. gondii* by both microscopy and semi-nPCR [44].

Interestingly, domestic cats can excrete over 20 million oocysts within 4 to 13 days post-infection with *T. gondii* [46]. To investigate earlier detection, 11 fecal samples were collected from infected cats on the third day post-infection. These samples were initially examined using microscopy, which failed to detect any *T. gondii* oocysts. Therefore, the single-tube method, along with semi-nPCR, was applied to determine whether these techniques could detect *T. gondii* at an earlier stage of infection in cats.

## Results

### Workflow and principle of the LAMP-CRISPR/Cas12b single-tube method

**Fig. 1:**
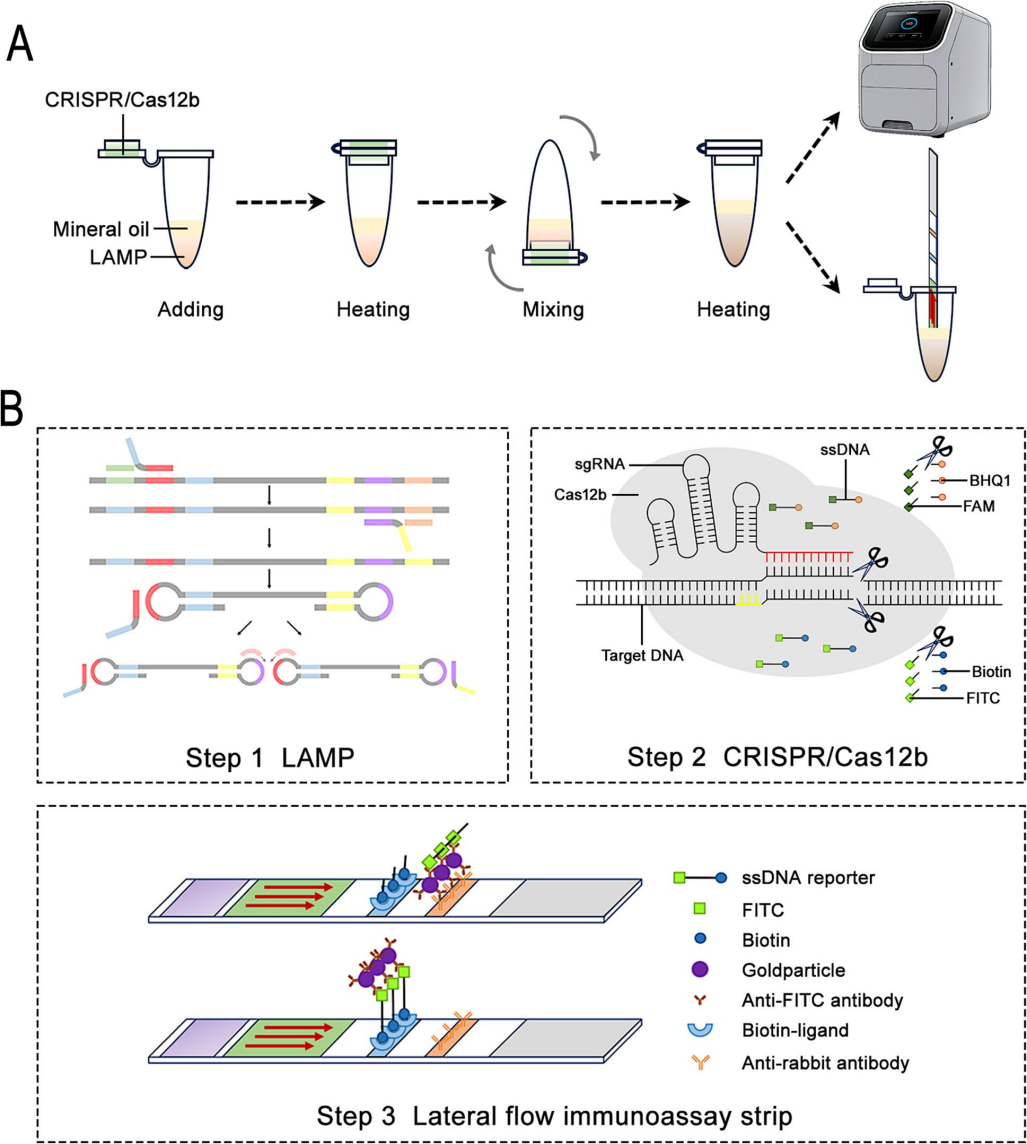
Detection mechanisms of the LAMP-CRISPR/Cas12b single-tube method. **A** Step-by-step operational workflow of the detection process. **B** Functional principles of each component in the LAMP-CRISPR/Cas12b system

The workflow and underlying principles of the LAMP-CRISPR/Cas12b single-tube method are shown in Fig. 1A, B. This innovative approach was used to target the *T. gondii B1* gene using LAMP primers designed to amplify sequences containing Protospacer Adjacent Motif (PAM) sites recognizable by CRISPR/Cas12b.

During detection, the Cas12b protein, guided by a specific sgRNA, binds to the target DNA sequence in the LAMP products. This binding activates Cas12b’s trans-cleavage function, which cleaves a surrounding free ssDNA reporter molecule. The reporter was labelled with a fluorescent tag (FAM) at one end and a quencher (BHQ1) at the other. Upon cleavage, the separation of these labels allows fluorescence detection via the QuantStudio 5 Real-Time PCR System.

In addition to fluorescence detection, results can be visually observed using a lateral flow immunoassay strip. For this, the ssDNA reporter was modified with FITC and biotin labels. The Milenia® HybriDetect lateral flow strip (Milenia Biotec, Gießen, Germany) employs gold nanoparticles for visualization. The strip contains two detection lines: the first line is coated with biotin-ligand, and the second line with anti-rabbit antibodies.

In negative samples, the intact ssDNA reporter binds to the biotin-ligand at the first detection line, generating a signal. For positive samples, the cleaved reporter enables the FITC-label to bind with anti-FITC antibodies conjugated to gold nanoparticles. This interaction produces a signal at the second detection line while reducing the signal at the first line, enabling clear differentiation between positive and negative results.

### Evaluation of LAMP for detection of *T. gondii*

LAMP plays a critical role in detecting target genes and amplifying signals. The reaction produced DNA products of varying lengths and structures, including linear and stem-loop forms, which were initially visualized using agarose gel electrophoresis. However, the resulting ladder-like banding pattern made it challenging to assess primer efficiency. To identify the most effective primers, real-time fluorescence signals were monitored using the QuantStudio 5 Real-Time PCR System with a fluorescent dye-enhanced LAMP system.

**Fig. 2:**
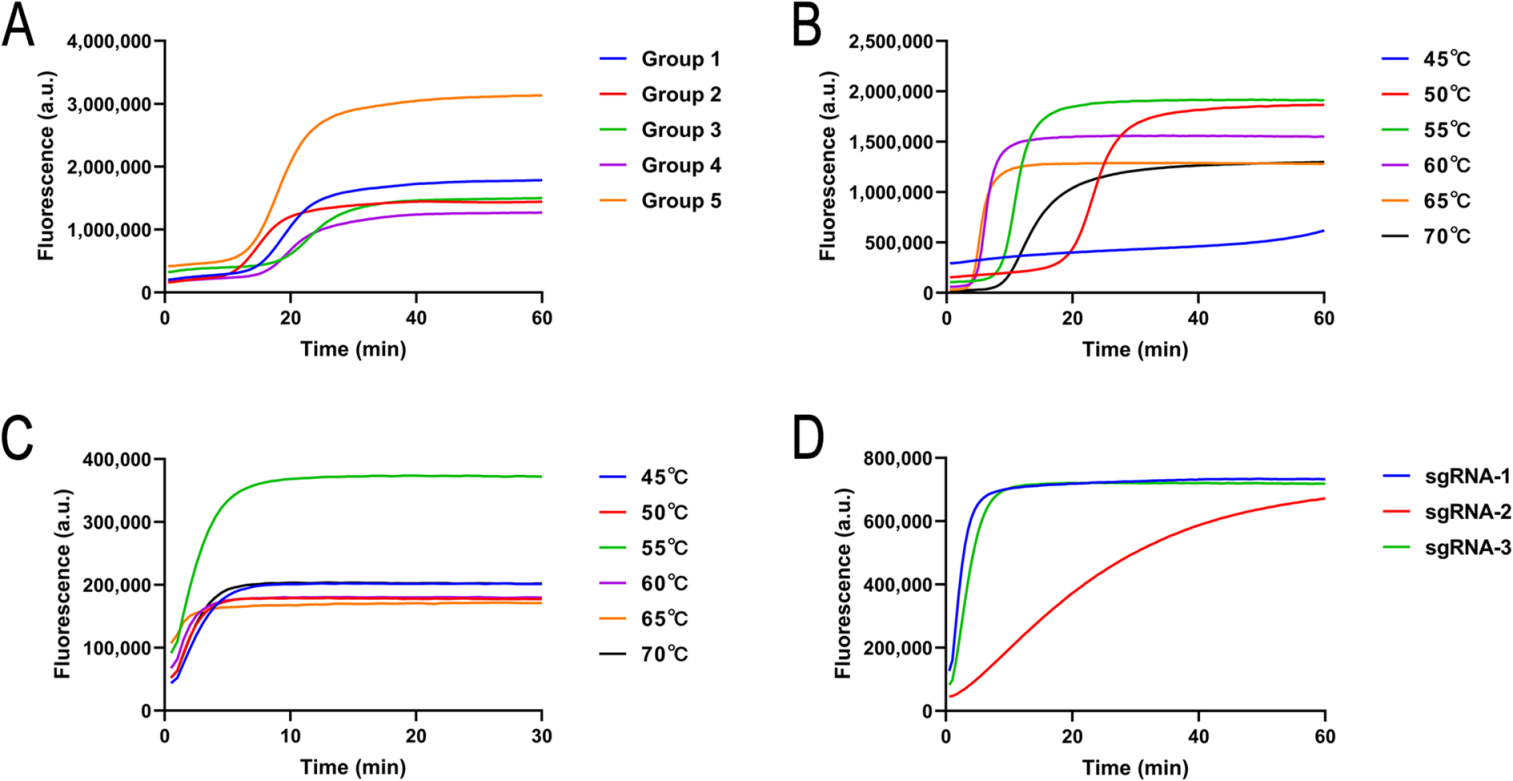
Optimization results of the LAMP and CRISPR/Cas12b systems. **A** Screening results for LAMP primers. **B** Optimization of LAMP reaction temperature. **C** Optimization of conditions for the CRISPR/Cas12b detection system. **D** Screening results of sgRNAs

As shown in Fig. 2A, all primer sets successfully facilitated LAMP reaction. Interestingly, group 5 primers outperformed others, completing amplification the fastest and producing significantly higher fluorescence intensity. Based on these results, group 5 primers were chosen for further experiments.

LAMP reactions were tested at various temperatures, with successful amplification observed at all temperatures except 45 °C (Fig. 2B). Since the LAMP-CRISPR/Cas12b single-tube method requires a shared reaction temperature for both LAMP and CRISPR/Cas12b, 55 °C was chosen as the operational temperature based on the CRISPR/Cas12b system’s operational requirements (Fig. 2C). At 55 °C, the LAMP reaction was completed in 10 min, with an amplification duration of 20 min set to ensure sufficient signal generation. For samples with lower DNA concentrations, the reaction time could be extended to improve detection sensitivity.

### Evaluation of the CRISPR/Cas12b system

To establish an effective CRISPR/Cas12b detection system, we designed sgRNAs specifically targeting the LAMP products. Since Cas12b relies on precise sgRNA guidance for target binding and cleavage, extensive optimization and validation were required. Fluorescence results using the FAM-12N-BHQ1 reporter (Fig. 2D) showed that both sgRNA-1 and sgRNA-3 effectively directed Cas12b for trans-cleavage. However, when these sgRNAs were tested with the FITC-12N-Biotin reporter on lateral flow immunoassay strips, sgRNA-3 demonstrated superior performance, producing stronger chromogenic results than sgRNA-1.

Fluorescence monitoring identified 55 °C as the optimal temperature for the CRISPR/Cas12b system (Fig. 2C). At this temperature, the system completed the cleavage reaction within 10 min, demonstrating its suitability for rapid diagnostic application.

### Development and evaluation of the LAMP-CRISPR/Cas12b single-tube method

**Fig. 3:**
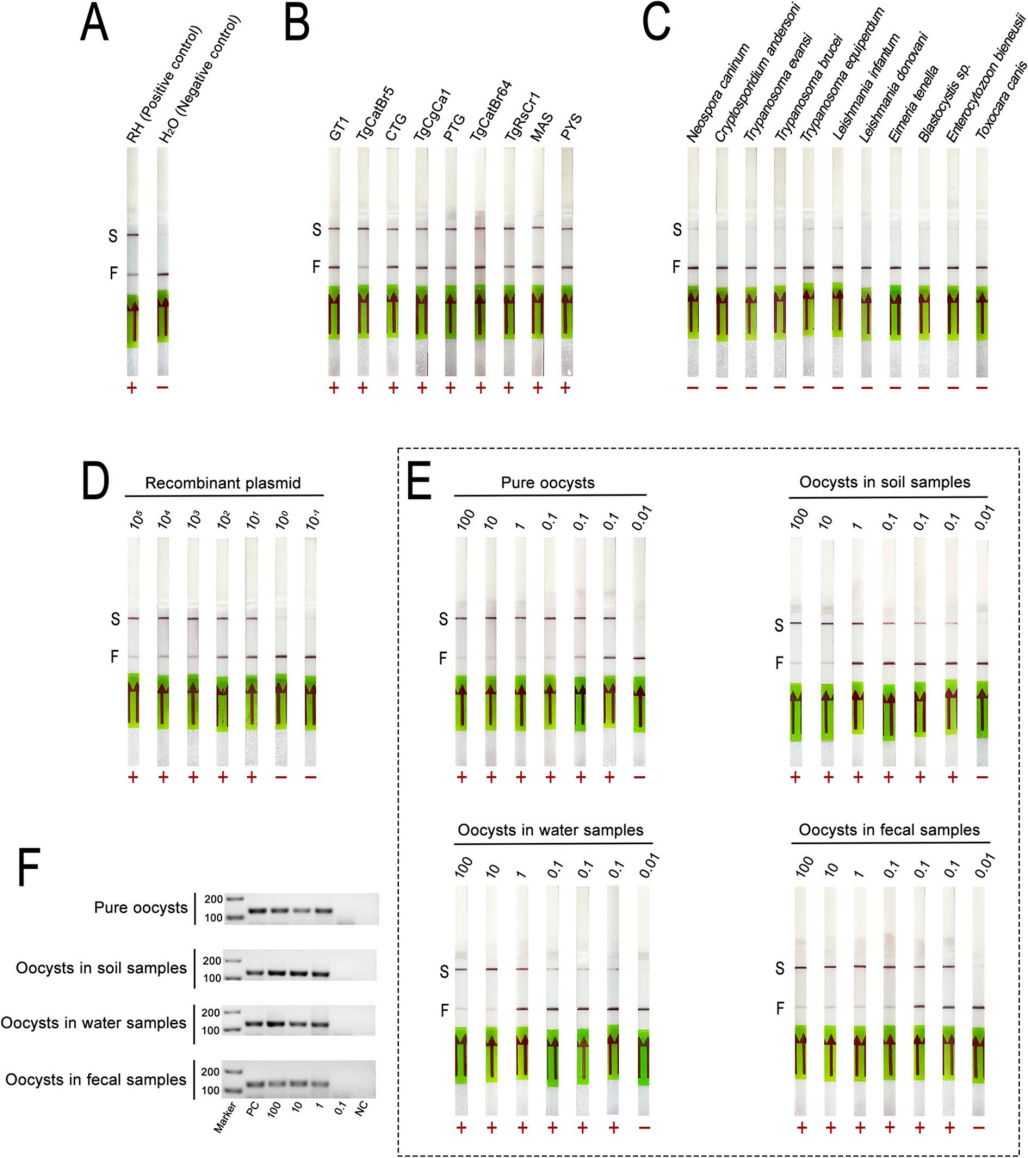
Evaluation of the LAMP-CRISPR/Cas12b single-tube method’s broad-spectrum capability, specificity, and sensitivity. **A** Positive and negative control detection results. **B** Evaluation of broad-spectrum detection with different *T. gondii* strains. **C** Specificity assessment of the method. **D** Sensitivity analysis based on plasmid concentration. **E** Sensitivity analysis based on oocyst counts. **F** Gel electrophoresis results from the semi-nPCR sensitivity test using oocyst counts. *F: First detection line; S: Second detection line

*Toxoplasma gondii* RH strain and nuclease-free water were used as standard detection samples. A visible signal on the first detection line denoted a negative control, while an enhanced second detection line with a weakened first line indicated a positive control (Fig. 3A). The method was tested on ten DNA samples from nine distinct *T. gondii* genotypes, successfully detecting all samples, and demonstrating its robustness and broad-spectrum capability (Fig. 3B). Additionally, it accurately distinguished *T. gondii* from eleven non-target parasites, highlighting its specificity (Fig. 3C).

Sensitivity was evaluated at both molecular and practical levels. At the molecular level, detection was assessed using a tenfold serial dilution of recombinant plasmid DNA of the *T. gondii B1* gene, with concentrations ranging from 1 × 10^5^ to 1 × 10^–1^ copies/µL. The single-tube method successfully detected plasmid concentrations of 10 copies/µL or higher (Fig. 3D).

For comparison, qPCR, a widely recognized method for its specificity and sensitivity, has a reported limit of detection (LOD) of 5 copies/µL for *T. gondii* surface water samples [16]. While the LAMP-CRISPR/Cas12b single-tube method offers comparable sensitivity, it eliminates the need for complex procedures and sophisticated equipment required by qPCR.

To validate the practical applications, the sensitivity of the method was tested using *T. gondii* oocysts in diverse environmental matrices, including cat feces, water, and soil. The method successfully detected as few as 0.1 oocyst, consistently yielding positive or weakly positive results in repeated tests (Fig. 3E). In contrast, conventional semi-nPCR had the detection limit of one oocyst (Fig. 3F). Nested PCR, a standard in epidemiological studies, similarly reports a detection threshold of one oocyst [47]. These findings underscore the LAMP-CRISPR/Cas12b single-tube method’s superior sensitivity and practicality, particularly for field applications and resource-limited settings.

### Application to environment samples

**Fig. 4:**
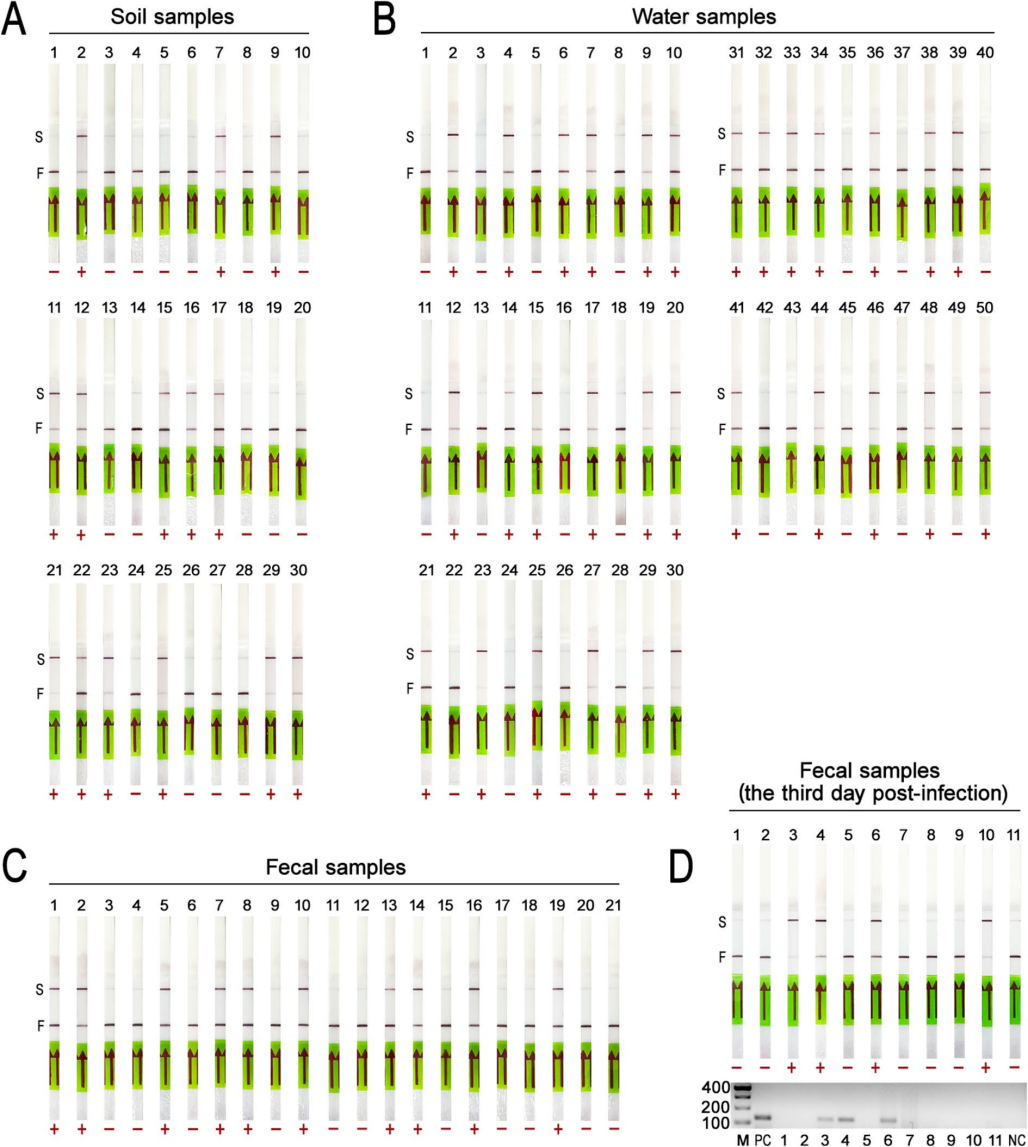
Environmental sample detection using the LAMP-CRISPR/Cas12b single-tube method. **A** Soil sample detection: 14 positives and 16 negatives. **B** Water sample detection: 30 positives and 20 negatives. **C** Cat fecal sample detection: 10 positives and 11 negatives. **D** Comparison of the LAMP-CRISPR/Cas12b and semi-nPCR in detecting *T. gondii* in 11 cat fecal samples collected 3 days post-infection. *F: First detection line; S: Second detection line

The reliability of the LAMP-CRISPR/Cas12b single-tube method was evaluated using 112 environmental samples. Among 30 soil samples, 14 tested positive (46.7%) and 16 were negative (53.3%) (Fig. 4A). For 50 water samples, 30 were positive (60%) and 20 negative (40%), aligning with established positive rates (Fig. 4B). Similarly, 21 cat fecal samples yielded 10 positive results (47.6%) and 11 negatives (52.4%), demonstrating consistency with semi-nPCR results (Fig. 4C). These outcomes validate the method’s effectiveness across environmental samples.

To assess early detection capability, 11 cat fecal samples were examined for *T. gondii* DNA 3 days post-infection. The single-tube method detected four positive samples (36.4%) (Fig. 4D), outperforming semi-nPCR which identified three positives (27.3%), and conventional microscopy which failed to detect any *T. gondii* oocysts. These findings highlight the single-tube method’s superior sensitivity and ability to facilitate earlier detection of *T. gondii* compared to traditional microscopy.

## Discussion

Traditional methods, such as microscopy and semi-nPCR, have limitations. Microscopy suffers from low sensitivity, while semi-nPCR requires expensive equipment, making it impractical in resource-limited settings. To address these challenges, we developed a novel LAMP-CRISPR/Cas12b single-tube method for rapid detection of *T. gondii* in environmental samples. This approach combines simplicity, efficiency, accuracy, and cost-effectiveness, making it an ideal tool for environmental monitoring in resource-constrained environments [39, 48].

LAMP offers significant advantages due to its speed, high sensitivity, and ability to operate at a constant temperature, making it especially suitable for resource-limited settings. It is also more resistant to inhibitors than other nucleic acid amplification techniques, enhancing its applicability to both clinical and environmental samples. However, the high amplification potential of LAMP can lead to contamination and false positives. To mitigate this, we incorporated Uracil-DNA Glycosylase (UDG) and a closed reaction system. The CRISPR/Cas12b system enhances detection by targeting specific DNA sequences and cleaving non-specific ssDNA reporters, improving signal amplification and sensitivity even in complex sample matrices. However, the system’s reliance on PAM sequences may limit its sensitivity and applicability to certain genomic regions.

We integrated the LAMP and CRISPR/Cas12b systems into a single tube method for detecting *T. gondii* in environmental samples. This streamlined approach eliminates the need for temperature cycling and sophisticated equipment, reducing contamination risks and making it particularly suitable for resource-limited settings. The single-tube method demonstrated exceptional sensitivity, being ten times more sensitive than conventional nPCR. However, potential errors during oocyst counting, gradient dilution, and DNA extraction should be considered, and the sensitivity results should be interpreted as preliminary reference data.

Our findings, along with studies such as Zhao et al. [49], support the effectiveness of CRISPR-Cas-based nucleic acid detection for identifying *T. gondii* in cat feces. This technology shows considerable potential for various environmental applications in parasitology. For example, CRISPR/Cas12a-based fluorescent signal amplification has been successfully used to detect *Cryptosporidium* in water samples, improving water quality monitoring [50].

In epidemiology, LAMP-CRISPR/Cas12b has proven effective in detecting drug-resistant strains of *Plasmodium falciparum*, aiding in treatment selecting and preventing the spread of resistant malaria parasites [51]. Similarly, CRISPR-Cas12a amplification supports high-sensitivity diagnosis of *Leishmania* infections at both the genus and subgenus levels, using DNA from human lesion samples [52]. This technology is also valuable for detecting *Cyclospora cayetanensis* in contaminated agricultural water, ensuring food and water safety [53].

In biodiversity and conservation research, CRISPR-based diagnostics can assess parasite loads in endangered species, supporting conservation efforts. Its simplicity and affordability make it ideal for resource-limited settings, as shown by its use in diagnosing *Schistosoma* infections via the CRISPR-Cas13a SHERLOCK platform [54]. Additionally, CRISPR/Cas12a enables field monitoring of plant parasitic nematodes, supporting sustainable agricultural practices [55]. In aquaculture, CRISPR-Cas12-based detection technology provides on-site monitoring of microsporidian parasites causing hepatopancreatic microsporidosis in aquatic animals, enabling timely disease control [56]. These diverse applications demonstrate the versatility and potential impact of LAMP-CRISPR/Cas12b technology in improving public health, environmental safety, and conservation. It offers a valuable tool for addressing the global challenge of parasitic zoonoses. Future studies should explore its clinical and epidemiological applications for toxoplasmosis surveillance and control.

## Conclusions

We have developed a novel single-tube method that combines LAMP with CRISPR/Cas12b and lateral flow immunoassay strips for the rapid, specific, sensitive and visual detection of *T. gondii* in environmental samples. This method is highly relevant to public health, providing a practical tool for monitoring *T. gondii* contamination in environmental settings. This study lays the groundwork for the widespread application of a CRISPR/Cas12b-based detection method in *T. gondii* epidemiological research. With further optimization of the primers, this method could be extended to detect other parasites and zoonotic pathogens in the future.

## Supplementary Information

Additional file 1: Table S1. Primer sequences of LAMP and sgRNA.

## Data Availability

The datasets supporting the findings of this article are included within the paper and its supplementary materials.

## Abbreviations

LAMP: Loop-mediated isothermal amplification
CRISPR: Clustered regularly interspaced short palindromic repeats
FAM: 6-Carboxyfluorescein
BHQ1: Black Hole Quencher-1
FITC: Fluorescein isothiocyanate
PCR: Polymerase chain reaction
qPCR: Quantitative real-time PCR
nPCR: Nested PCR
ssDNA: Single-stranded DNA
sgRNA: Single-guide RNA
