# Supplementary material for: A novel single-tube LAMP-CRISPR/Cas12b method for rapid and visual detection of zoonotic Toxoplasma gondii in the environment
Source: Infect Dis Poverty. 2024 Dec 10;13:94. doi: 10.1186/s40249-024-01266-5 (PMC11629535; doi:10.1186/s40249-024-01266-5)
Supplement: Supplementary file 1 — Additional file 1: Table S1. Primer sequences of LAMP and sgRNA. [file 40249_2024_1266_MOESM1_ESM.pdf]

Additional file 1: Table S1. Primer sequences of LAMP and sgRNA.

Number	Primer name	Primer sequence (5'-3')
Group 1	FIP	GTGGTCGACTTCATGGGACGATTCTGTTCTGTTCGCTGTCTG
	BIP	TCACTGTCACGTACGACATCGCGGCGGACCTCTCTTGTCT
	F3	GTCCGTCGTAATATCAGGCC
	B3	TTCTTCAGCCGTCTTGTGG
	Loop F	GCAGTAAGGGTGCCCTAGA
	Loop B	ATTCAAGGGAAGAGATCCAGCA
Group 2	FIP	CTTCAGCCGTCTTGTGGGGGGATCCAGCAGATCTCGTTCG
	BIP	TTGTGCTGCCTCCTCTCATGGCAGCGGGAAATACAGCTCTT
	F3	CGACATCGCATTCAAGGGAA
	B3	GTGGCTTTTCTGGAGGTACA
	Loop F	GGACCTCTCTTGTCTCGAATACA
	Loop B	CAAATGCCAGAAGAAGGGTACGTGT
Group 3	FIP	CTTCAGCCGTCTTGTGGGGGCAAGGGAAGAGATCCAGCAG
	BIP	TGCCTCCTCTCATGGCAAATGCCTGTATTTGCCAGCGGGAA
	F3	TGTCACGTACGACATCGCA
	B3	GTGGCTTTTCTGGAGGTACA
	Loop F	CTTGTCTCGAATACACGAACGAG
	Loop B	CGTGTTGCATCATAACAAGAGCTGT
Group 4	FIP	GTGGTCGACTTCATGGGACGATTCGCTGTCTGTCTAGGGC
	BIP	TCACTGTCACGTACGACATCGCGGCGGACCTCTCTTGTCT
	F3	GTCCGTCGTAATATCAGGCC
	B3	TTCTTCAGCCGTCTTGTGG
	Loop F	CAAATACTTCTCTTGCAGTAAGGGT
	Loop B	ATTCAAGGGAAGAGATCCAGCA
Group 5	FIP	TTCTCTTGCAGTAAGGGTGCCCCGTCCGTCGTAATATCAGGC
	BIP	ATCGTCCCATGAAGTCGACCACTGCTGGATCTCTTCCCTTGA
	F3	CGTGGATTTCCGTTGGTTCC
	B3	GGCGGACCTCTCTTGTCT
	Loop F	CAGACAGCGAACAGAACAGAAG
	Loop B	TGTTTCCTCTCTTCACTGTCACG
sgRNA	Spacer	GAAATTAATACGACTCACTATAGGGGTCTAGAGGACAGAATTTTTCAACGGG TGTGCCAATGGCCACTTTCCAGGTGGCAAAGCCCGTTGAGCTTCTCAAATCT GAGAAGTGGCAC
	T7-sgRNA-F	GAAATTAATACGACTCACTATAGGG
	T7-sgRNA-R1	TCACCTGTATTTGCCAGCGGGTGCCACTTCTCAGATTTGAGAAG
	T7-sgRNA-R2	CATGAGAGGAGGCAGCACAAGTGCCACTTCTCAGATTTGAGAAG
	T7-sgRNA-R3	GCGATGTCGTACGTGACAGTGTGCCACTTCTCAGATTTGAGAAG
